# Research advances on molecular mechanism and natural product therapy of iron metabolism in heart failure

**DOI:** 10.1186/s40001-024-01809-4

**Published:** 2024-04-24

**Authors:** Tianqing Zhang, Li Luo, Qi He, Sijie Xiao, Yuwei Li, Junpeng Chen, Tao Qin, Zhenni Xiao, Qingliang Ge

**Affiliations:** 1 Department of Cardiology, Changde Hospital, Xiangya School of Medicine, Central South University, Hunan, China; 2 People’s Hospital of Ningxiang City, Ningxiang City, China

**Keywords:** Iron deficiency, Iron overload, Heart failure, Antioxidant, Mitochondria

## Abstract

The progression of heart failure (HF) is complex and involves multiple regulatory pathways. Iron ions play a crucial supportive role as a cofactor for important proteins such as hemoglobin, myoglobin, oxidative respiratory chain, and DNA synthetase, in the myocardial energy metabolism process. In recent years, numerous studies have shown that HF is associated with iron dysmetabolism, and deficiencies in iron and overload of iron can both lead to the development of various myocarditis diseases, which ultimately progress to HF. Iron toxicity and iron metabolism may be key targets for the diagnosis, treatment, and prevention of HF. Some iron chelators (such as desferrioxamine), antioxidants (such as ascorbate), Fer-1, and molecules that regulate iron levels (such as lactoferrin) have been shown to be effective in treating HF and protecting the myocardium in multiple studies. Additionally, certain natural compounds can play a significant role by mediating the imbalance of iron-related signaling pathways and expression levels. Therefore, this review not only summarizes the basic processes of iron metabolism in the body and the mechanisms by which they play a role in HF, with the aim of providing new clues and considerations for the treatment of HF, but also summarizes recent studies on natural chemical components that involve ferroptosis and its role in HF pathology, as well as the mechanisms by which naturally occurring products regulate ferroptosis in HF, with the aim of providing reference information for the development of new ferroptosis inhibitors and lead compounds for the treatment of HF in the future.

## Introduction

Heart failure (HF) is a severe end-stage disease caused by various factors such as infection, fatigue, high blood pressure, myocardial ischemia, arrhythmias, and heart overload [1]. Epidemiologic studies have shown that by 2030, the prevalence of heart failure in the United States is expected to increase by 46%, reaching 8 million adults, and treatment costs will increase by 127%, reaching 697 billion US dollars, which amounts to approximately 244 US dollars per American adult [2, 3]. Currently, China has approximately 15 million patients with heart failure, and with the aging of the population, an increase in cardiovascular survivorship, and the lack of timely treatment of diseases such as diabetes, chronic kidney disease, and other chronic diseases, the incidence of heart failure is increasing [4]. Currently, medical treatment is still the foundation of heart failure treatment, and classic basic drugs such as positive inotropic drugs, diuretics, angiotensin-converting enzyme inhibitors, angiotensin receptor blockers, beta-receptor blockers, and aldosterone receptor antagonists are used in the treatment of heart failure. However, they cannot completely prevent the development of heart failure [5, 6]. In addition, ultrafiltration treatment, heart resynchronization therapy (CRT), implantable cardioverter defibrillation (ICD), left ventricular assist device (LVAD), heart transplantation, coronary angiography, and other heart interventional therapies, catheter ablation, stem cell treatment, and gene therapy have been further developed [2], but their use is still relatively low [7, 8]. China still faces significant challenges in the prevention and control of heart failure, and the development of new targets and mechanisms for the occurrence and development of heart failure is expected to help reduce the readmission rate and mortality of heart failure.

Ferroptosis is a form of cell programmed death that is regulated by ferredoxin-dependent iron metabolism and is triggered by the toxicity of lipid peroxidation products on the cell membrane [9]. Research has shown that this unique cell death mechanism is driven by phospholipid peroxidation dependent on iron and is regulated by various cell metabolic pathways (including oxidative stress, iron metabolism, mitochondrial activity, amino acid, lipid, and glucose metabolism) and various signal pathways related to disease [10]. The Xc-–GSH–GPX4 pathway is the main route for regulating ferroptosis. The Xc-system can exchange cysteine and glutamate intracellularly and extracellularly, and is composed of a homodimer of the anion transporter family member 3 member 2 (SLC3A2) and SLC7A11 [11]. Through the Xc-system, cysteine is transported into the cell for GSH synthesis, while glutamate within the cell can be transported out of the cell [12]. GSH has antioxidant properties and can participate in the generation of glutathione peroxidase 4 (GPX4) [13]. GPX4 is an antioxidant enzyme that maintains the dynamic balance of the cell oxidative/ reductive state, mainly as a critical endogenous antioxidant inhibiting phospholipid peroxidation, with upstream GSH as its substrate [14]. Therefore, targeted inhibition of the Xc-system can lead to GSH depletion, GPX4 inactivation, promotion of malondialdehyde (MDA) production, and the accumulation of lipid ROS, resulting in ferroptosis [15, 16]. The commonly used ferroptosis inducer erastin can directly inhibit the Xc-system, consume cysteine, and inhibit GSH synthesis, thereby promoting cell ferroptosis [17]. The ferroptosis inducer RSL3 acts directly on cysteine to inhibit GPX4, promoting the accumulation of lipid ROS and causing ferroptosis [14].

The abnormal changes in iron content in the latest cardiomyocytes can have a significant negative impact on heart function and exacerbate heart failure (HF) [18]. Research has shown that iron deficiency is a typical feature of HF [19]; iron overload can cause tissue damage due to oxidative stress, and severe iron overload can cause refractory HF [20]. Therefore, targeting iron metabolism may become a key factor in treating HF and improving prognosis. Although the precise mechanism by which abnormal regulation of iron metabolism mediates HF has not been fully explained, targeted protection drugs for HF are gradually being developed, especially focusing on natural compounds that regulate HF iron death and iron metabolism [21, 22]. These natural compounds may provide ligands or core structures for future drug development targeting iron-related death processes, such as ferroptosis. Therefore, this study will not only provide a review of the normal iron metabolism process, summarize the potential mechanisms of iron metabolism disorders and HF, and offer new ideas and strategies for HF treatment and prognosis, but also review the currently reported natural compounds that regulate HF ferroptosis.

## Overview of iron metabolism

Iron is the most abundant essential trace element in the human body, widely distributed throughout the various tissues and organs of the body. As a component of ferric-sulfur clusters enzymes [such as cytochromes, nicotinamide adenine dinucleotide phosphate (NADPH), and succinate dehydrogenase], iron catalyzes the transport of oxygen, generates energy through oxidizing phosphorylation, and participates in the construction of enzymes that generate peroxides and nitric oxide, playing a crucial role in physiological and pathological processes. The body maintains the stability of iron ions by continuous absorption, circulation, utilization, excretion, and fine regulatory mechanisms.

### Iron uptake, circulation, storage and excretion

#### Iron uptake and excretion

According to the different absorption mechanisms, iron in the diet can be divided into heme iron and non-heme iron [23]. Heme iron is mainly present in animal foods and is released from hemoglobin and myoglobin in meat foods. Non-heme iron is mainly present in plant foods. Heme iron has better bioavailability than non-heme iron. The specific absorption mechanism of heme iron is not very clear, and it may enter enterocytes through heme carrier protein 1 (HCP1) on the duodenal epithelium [24], releasing iron from heme by heme oxygenase-1 (HO-1) [25]. Three-valent iron in non-heme iron needs to be reduced to ferrous iron by ferredoxin-like proteins (FDPs) at the brush border of the intestine, then absorbed by divalent metal transporter 1 (DMT1) and transported into the cell. In the blood, iron is mainly complexed with transferrin (Tf), which is mainly internalized through high-affinity Tf receptors on the surface of cells [10]. An acidic environment in early lysosomes promotes the release of iron from Tf, which is then reduced to ferrous iron by the prostatic six-transmembrane epithelial antigen of the prostate 3 (STEAP3) and transported into the cell cytoplasm through DMT1. The iron-free Tf/TfR1 complex returns to the cell surface and dissociates [26]. In addition to these mechanisms, iron can also enter cells through calcium channels and zinc transporters [27].

The excretion of cellular iron is achieved through the expression of membrane iron transporters (ferroportin, FPN) [28]. These are abundant in cells that maintain plasma iron levels (such as villous epithelial cells, macrophages, and hepatocytes) [26], and also play an important role in local iron regulation in other cells (such as cardiomyocyte). FPN is negatively regulated by ferrostatin, which has been shown to bind to FPN1 and cause its internalization and degradation [29].

#### Iron storage and recycling

Unutilized or output iron is stored in ferritin, which is primarily present in the liver. The majority of iron required for erythrocyte production is recycled by macrophages from aged or damaged erythrocytes. If there is a lack of supply, ferritinophagy, which is mediated by nuclear receptor coactivator 4 (NCOA4), is used to mobilize the stored iron from the liver [26, 30]. The physiological range of liver iron deposition is 300 mg to 1 g, but it can reach as high as 25 to 30 g in patients with hereditary hemochromatosis [31]. When the level of serum iron is at about 60% saturation beyond the buffering capacity of transferrin, non-transferrin-bound iron (NTBI) is present, which is imported into hepatocytes through solute carrier family 39 member 14 (SLC39A14) [32].

### Iron regulation

Maintaining the iron homeostasis in cells and organisms ensures sufficient iron supply and prevents the accumulation of toxic iron. At the cellular level, the expression and translation of proteins involved in iron metabolism are regulated by iron response elements (IREs) and iron regulatory proteins (IRPs). IRE-binding proteins, including ferritin, TfR1, DMT1, 5-aminolevulinate synthase 2 (ALAS2, involved in heme biosynthesis), and hypoxia-inducible factor 2α (HIF2α), regulate the expression of these proteins. IRP1 and IRP2 can recognize and bind to IREs with specific structures and sequences. When IRP1 or IRP2 binds to IREs in the 5' non-translated region, it blocks translation, while binding to IREs in the 3' non-translated region stabilizes mRNA and prevents endonuclease degradation. When the intracellular iron level is low, IRPs lack the necessary iron–sulfur cluster for binding to IREs, and thus, binding to IREs can suppress ferritin translation and stabilize TfR1 mRNA, leading to decreased iron storage and increased iron absorption [33]. As the concentration of iron ions in cells increases, iron is bound to the iron–sulfur cluster, and binding to IRPs can prevent IRP-IRE interactions. At the whole-body level, iron regulation is mainly dependent on hepcidin, a liver-derived sex steroid hormone that is an important regulator of iron homeostasis [34]. Hepcidin binds to FPN and internalized, followed by ubiquitination and transportation into the lysosomes for degradation [35]. This inhibition of iron export from cells prevents iron efflux. When the blood iron level increases, hepcidin secretion increases, and FPN expression on the cell surface decreases, leading to decreased iron efflux from cells and reduced serum and cellular fluid iron levels [36]. In contrast, low hepcidin levels increase iron efflux from hepatocytes and macrophages, leading to increased serum iron concentration and transferrin saturation. The deficiency or excess of hepcidin can lead to corresponding diseases. After acute myocardial infarction, hepcidin expression increases, and the specific loss of hepcidin in cardiomyocytes cannot improve heart function [37]. The decrease in hepcidin concentration or the reduction in hepcidin-FPN binding caused by hemochromatosis can lead to iron overload and extensive tissue damage [38]. Inflammatory responses also lead to an increase in hepcidin expression, and low iron levels are simultaneously observed [39]. Hepcidin responds mainly to increases in intracellular iron storage or inflammatory signals, and when iron overload occurs in hepatocytes, it can also suppress hepcidin production by inhibiting the BMP/SMAD and IL-6/STAT3 signaling pathways [40].

## Mechanism of ferroptosis

**Fig. 1:**
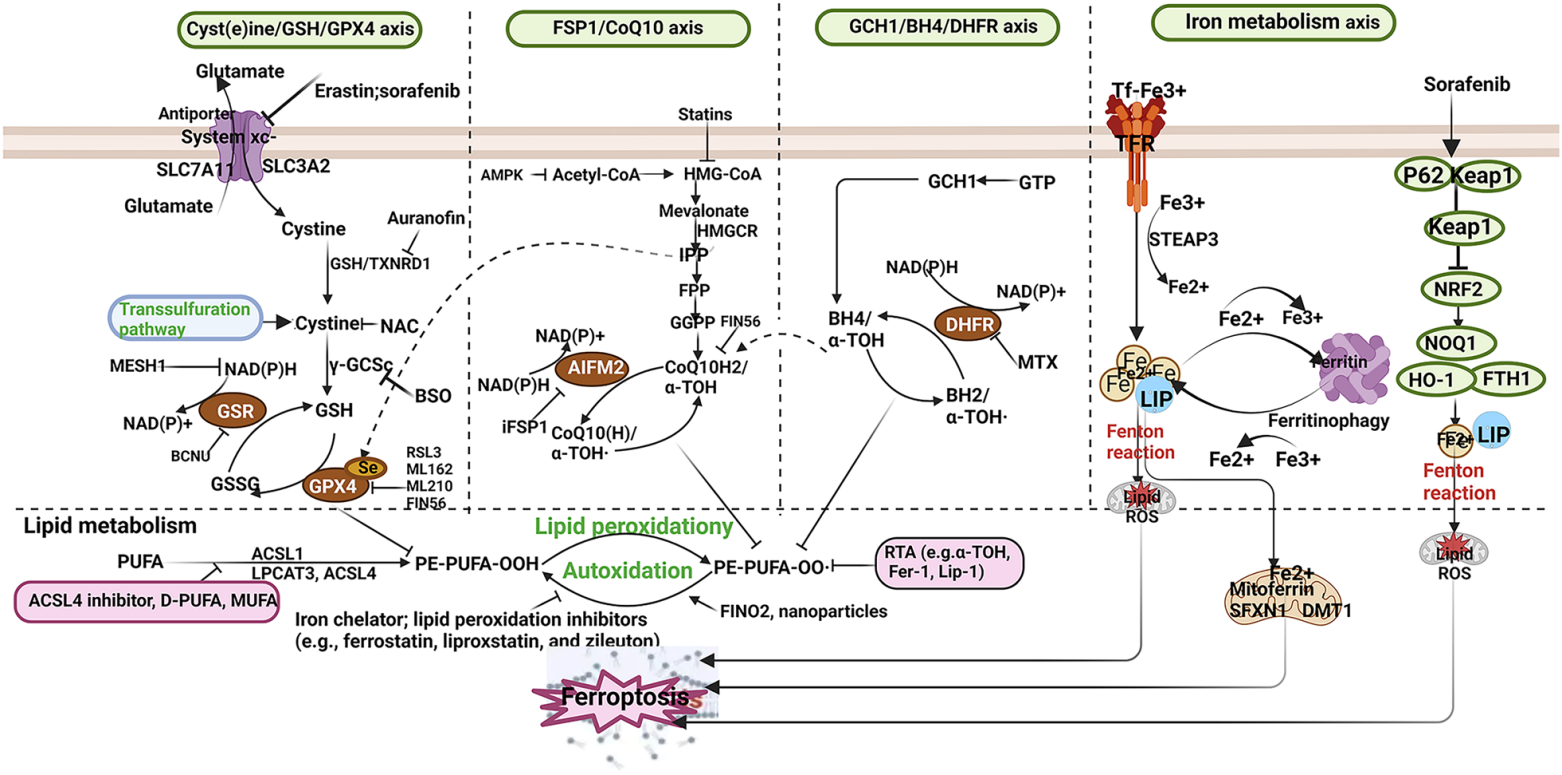
Summary of the mechanism of ferroptosis (GPX4: glutathione peroxidase 4; GSH: glutathione; SLC7A11: solute carrier family 7 member 11; SLC40A1: solute carrier family 40 member 1)

Ferroptosis is a novel type of cell death characterized by iron-dependent, nonapoptotic, and increased lipid ROS (ROS). It differs from traditional cell death in three aspects: (1) morphologically, mitochondria become smaller and cristae decrease, leading to increased membrane density and increased tendency to rupture; (2) biochemically, there is an overload of iron, reduced GSH synthesis or consumption, decreased activity of GSH peroxidase 4 (GPX4), and inhibited System Xc-, leading to a disruption of the oxidative–reductive balance and accumulation of lipid peroxides; (3) genetically, multiple effectors participate in ferroptosis and are regulated by multiple metabolic pathways [41, 42]. The mechanisms of ferroptosis include iron overload, lipid peroxidation, abnormal GSH–GPX4–ROS pathway, cancer suppressor gene P53 (P53), NADPH/ferroptosis inhibitory protein 1/coenzyme Q10 (NADPH/FSP1/CoQ10) pathway, and oxidative stress. These mechanisms lead to the accumulation of lipid ROS, destroying the redox balance in the body, leading to cell ferroptosis (Fig. 1).

### Iron overload

Iron is an essential trace element in human body and plays an important role in the function of cardiomyocytes. Two Fe3 + ions (Fe3 +) bind to one transferrin (Tf) on the surface of cardiomyocyte cytosol, and the transferrin receptor 1 (TFR1) on the membrane internalization transfers the Fe3 + ions into the cell [43]. Under physiological conditions, Fe3 + ions are reduced to Fe2 + ions by the metal reductase 3 (STEAP3) and then stored in the dynamic iron pool [44, 45]. The Fe2 + ions are then transported to the cytoplasm by the divalent metal transporter 1 (DMT1) and stored in the dynamic iron pool [46]. The dynamic iron pool can not only store Fe2 + ions but also store the required iron proteins. The ferric transport protein 1 (FPN1) is the only channel for Fe2 + ions to leave the cell. When there is an excess of Fe2 + ions, FPN1 starts to play an autocrine role, and some Fe2 + ions are stored in the heavy chain 1 (FtH1) and light chain 1 (FtL1) of ferritin. Experimental findings have shown that erastin and the GSH peroxidase 4 inhibitor (RSL3) can trigger ferroptosis. The mechanism involves increasing the expression of TFR1, enhancing the transport of Fe2 + ions, and reducing the regulation of FtH1 and FtL1, leading to further release of Fe2 + ions, resulting in the accumulation of large amounts of Fe2 + ions in the cell [47]. Fe2 + ions can generate many hydroxyl radicals and a large amount of ROS through the Fenton reaction and the Haber–Weiss reaction, which are harmful to the cell and lead to cell ferroptosis [48].

### Lipid peroxidation

Lipid peroxidation is one of the characteristics of ferroptosis, which is characterized by the accumulation of intracellular ROS that disrupts the oxidative–reductive balance and attacks cell membranes. The phospholipids in cell membranes are composed of lipid polyunsaturated fatty acids (PUFAs), which are easily oxidized. Recently, it has been reported that lipid peroxidation can damage the stability of the lipid bilayer and cause membrane disruption [49]. During the Fenton reaction, hydroxyl groups are produced, and the hydrogen in PUFAs is oxidized by hydroxyl groups to form lipid radicals [50]. Then, lipid radicals react with oxygen molecules to form a lipid peroxyl radical, which then reacts with nearby PUFAs to cause lipid peroxidation. This captures its hydrogen atoms and generates two new products called hydroperoxides and new lipid atomic groups, which lead to a chain reaction, generating many lipid peroxides, destroying the cell membrane, and causing ferroptosis [51]. 4-Hydroxynonenal (4-HNE) and malondialdehyde (MDA) are key biological biomarkers for verifying the occurrence of ferroptosis and lipid peroxidation. They can react with nucleic acids and proteins to further damage cells. In addition, Fe2 + can act as a cofactor of lipoxygenase (LOX) to catalyze the peroxidation of PUFAs, producing lipid peroxides [52]. It has been found that 15-LOX can be enzymatically hydrolyzed on PUFAs to produce large amounts of hydrogen peroxide, and 15-LOX is composed of arachidonic acid (AA) or arachidonic acid. It is mainly distributed on the membrane phospholipids of erythrocytes, and it can spontaneously cause peroxidation in the presence of hydroxyl radicals. To bind to the phospholipids on the membrane, PUFAs require the participation of phospholipase A2 long-chain family member 4 (PLCL4) and acyl-CoA synthetase long-chain family member 4 (ACSL4), as well as CoA. Therefore, lipid peroxidation-induced ferroptosis occurs. It has been reported that PLCL4 and ACSL4 participate in the peroxidation of PUFAs on the membrane and become biomarkers of ferroptosis [53].

### System Xc-/GSH/GPX4/ROS pathway exception

GSH and GPX4 play a key role in the process of ferroptosis. GSH is an essential antioxidant that is indispensable in ferroptosis, and it is mainly present in the cell in the reduced form (GSH) and the oxidized form (GSSG). GSH provides electrons to GPX4, which is used to degrade lipid peroxides. GPXs are enzymes that catalyze the degradation of peroxides [54, 55]. As members of the GPX family, GPX4 together with GSH degrades lipid peroxides and reduces cell damage [56]. In physiological conditions, cysteine residues located on the cell surface are transported into the cell by the cysteine-glutamate transporter (System Xc-), while glutamate is transported out of the cell [57]. The cysteine is then converted to cysteine sulfone. Under the catalysis of glutamate cysteine ligase and glutathione synthetase, cysteine sulfone and glutamate react to form GSH. GSH combined with GPX4 can significantly reduce the amount of excessive lipid peroxides and prevent ferroptosis. GPX4 inhibition of lipid peroxidation requires GSH assistance, and GSH synthesis depends on the decisive role of cysteine. When System Xc- transportation is inhibited, cysteine and glutamate cannot be transferred mutually, resulting in a decrease in intracellular cysteine production, followed by a decrease in GSH production, leading to an increase in the activity of GPX4, resulting in a decrease in the level of lipid peroxides and an increase in the occurrence of ferroptosis. Eratin can inhibit the cysteine-glutamate transporter and prevent the transportation of cysteine, resulting in a decrease in GSH production and an inhibition of GPX4 activity, leading to an increase in the antioxidant ability and the occurrence of ferroptosis [58]. As a ferroptosis inducer, RSL3 is different from Eratin in that it does not affect the GSH concentration, but can directly inhibit the activity of GPX4 due to the cysteine residue at the active site of GPX4 interacting with RSL3. In addition, flavin proteins (mitochondrial-related apoptosis inducer 2, MRE2) can protect GPX4 and prevent cell ferroptosis [59].

### Oxidative stress

Oxidative stress is an important mechanism underlying HF and also leads to ferroptosis in cells. Oxidative stress is primarily caused by an imbalance between oxidation and reduction, which is caused by the accumulation of lipid peroxides and the deficiency of antioxidants. An important signal pathway that regulates the oxidative stress response is the Nrf2/heme oxygenase-1 (HO-1) axis [60]. Nrf2 is a decisive factor in reducing lipid peroxidation, and some of Nrf2's target genes participate in the oxidative stress response and iron metabolism, regulating the System Xc- and GPX4, which play important roles in ferroptosis [61]. Nrf2 enhances the transport of System Xc- out of the cell, increases GSH synthesis, and increases the activity of GPX4, thereby strengthening the clearance of lipid peroxides, improving antioxidant ability, and suppressing cell ferroptosis. In animal experiments, activation of the Nrf2/antioxidant response element (Nrf2/ARE) signal pathway can increase the activity of superoxide dismutase (SOD) and glutathione peroxidases (GPXs) and further enhance heart function, reduce ROS and other harmful substances that are harmful to cells, and suppress cardiomyocyte ferroptosis [62, 63]. HO-1 plays a regulatory role in the degradation of heme. The products of HO-1 degradation include Fe2 + , which can accumulate excessively and thereby initiate ferroptosis. This is a major cause of heart damage caused by doxorubicin. In mice experiments, doxorubicin can regulate the upregulation of HO-1, iron overload, and lipid peroxidation, leading to ferroptosis. Administration of ferroptosis inhibitors can significantly alleviate the damage caused to the heart by doxorubicin, suppress HO-1 gene expression, and remove Nrf2-related factors. After removing Nrf2-related factors, the amount of iron overload in mouse cardiomyocytes caused by doxorubicin is reduced, thereby protecting heart function.

### NADPH/FSP1/CoQ10 pathway

In 2019, researchers abroad reported the discovery of a mitochondrial apoptosis inducer 2 (AIFM2), which revealed that AIFM2 overexpression can effectively protect cells and is not influenced by ferroptosis inducers. This study confirmed that AIFM2 can inhibit ferroptosis and redesignated AIFM2 as FSP1. Studies have shown that under normal conditions of GPX4 function, loss of FSP1 can lead to increased peroxidation of phospholipids and cause ferroptosis [64]. The key reason for the inhibition of ferroptosis by FSP1 is CoQ10. CoQ10 is a lipophilic antioxidant drug, and FSP1 catalyzes the reduction of CoQ10 to ubiquinone by NADPH, capturing lipid peroxides and reducing them, thereby reducing the accumulation of lipid peroxides and suppressing cell iron apoptosis [42]. NADPH/FSP1/CoQ10 is a pathway with the same inhibitory effect on ferroptosis as GSH/GPX4.

### Tumor suppressor P53

Since its discovery in 1979, p53 has remained a focus of interest in the field of oncological research [65]. In fact, p53 has important functions beyond cancer (such as development, stem cells, and some non-cancer diseases) [66]. p53 can be inducible by a wide range of intracellular/extracellular stimuli and pressures (such as DNA damage, oncogenic activation, ribosomal or telomere-related stresses, and nutrient deprivation), and acts as a regulator of a diverse set of downstream genes to produce various effects at the cellular and organismal levels (such as cell cycle arrest, DNA repair, senescence, apoptosis, and ferroptosis), thereby helping cells/organisms to resist stimuli. p53 can also function without depending on its transcriptional activity [67]. p53 primarily functions as a transcriptional regulator, activating or suppressing the transcription of multiple downstream target genes. The functions of these target genes mainly include inducing cell cycle arrest, DNA repair, regulating cell metabolism, cell senescence, cell apoptosis, and recently discovered inducing cell ferroptosis [68].

In 2015, JIANG et al. discovered a tumor suppressor gene P53, which participated in the regulation of multiple cells and was possibly achieved by inhibiting the expression of solute carrier family 7 member 11 transporter (SLC7A11), a subunit of the SLC7A11, on the cysteine-glutamic acid transporter receptor, thereby inhibiting the transport of cysteine into the cell, reducing the activity of glutathione peroxidase 4 (GPX4) to reduce lipid peroxides, and ultimately inducing cell ferroptosis [69]. This was the first discovery of P53 suppressing SLC7A11 expression at the transcriptional level to promote ferroptosis and contribute to tumor suppression. Further research showed that acetylation of P53 K101 played an important role in suppressing SLC7A11 expression. Interestingly, the P53 3KR mutant retained the ability to induce ferroptosis while losing the ability to induce cell cycle arrest, senescence, or apoptosis. However, the P53 4KR mutant and a 非洲人来源 p53 SNP P47S lost the ability to induce ferroptosis and tumor suppression. These results indicate that ferroptosis induction may be the most important weapon of P53 in suppressing tumors. The authors further discovered that P53 could promote SAT1 expression to enhance the function of another member of the ALOX family, ALOX15, to enhance ferroptosis [70]. In addition, the p53-SLC7A11 axis can also promote ferroptosis through a GSH-independent mechanism. They found that the lipid peroxidase ALOX12 was a key regulatory factor in p53-dependent ferroptosis. However, SLC7A11 directly interacted with ALOX12 to limit its function. When P53 downregulated SLC7A11, ALOX12 was released. Free ALOX12 could oxidize the fatty acid chain of membrane phospholipids, leading to ferroptosis [71]. A recent study found that phospholipase iPLA2β was an important factor in regulating ferroptosis induced by high levels of ROS [72]. Research also showed that P53 could regulate PHGDH to inhibit serine synthesis, which may affect GSH synthesis to promote ferroptosis [73]. Additionally, P53 could promote lncRNA PVT1 expression or directly interact with the mitochondrial iron transporter SLC25A28 to promote ferroptosis [74]. The two markers of ferroptosis, PTGS2 and CBS, were also proven to be targets of P53. These findings support the hypothesis that P53 promotes ferroptosis. In addition to SLC7A11, many other P53 target genes also have the ability to promote iron death, and the pathological mechanisms often involve increased GSH consumption and polyunsaturated fatty acid peroxidation [68]. Moreover, in a rat heart infarction model, it was found that proteasome-specific protease 7 (PSMC7) could activate the P53/TFR1 pathway to enhance iron uptake, leading to ferroptosis [75].

**Fig. 2:**
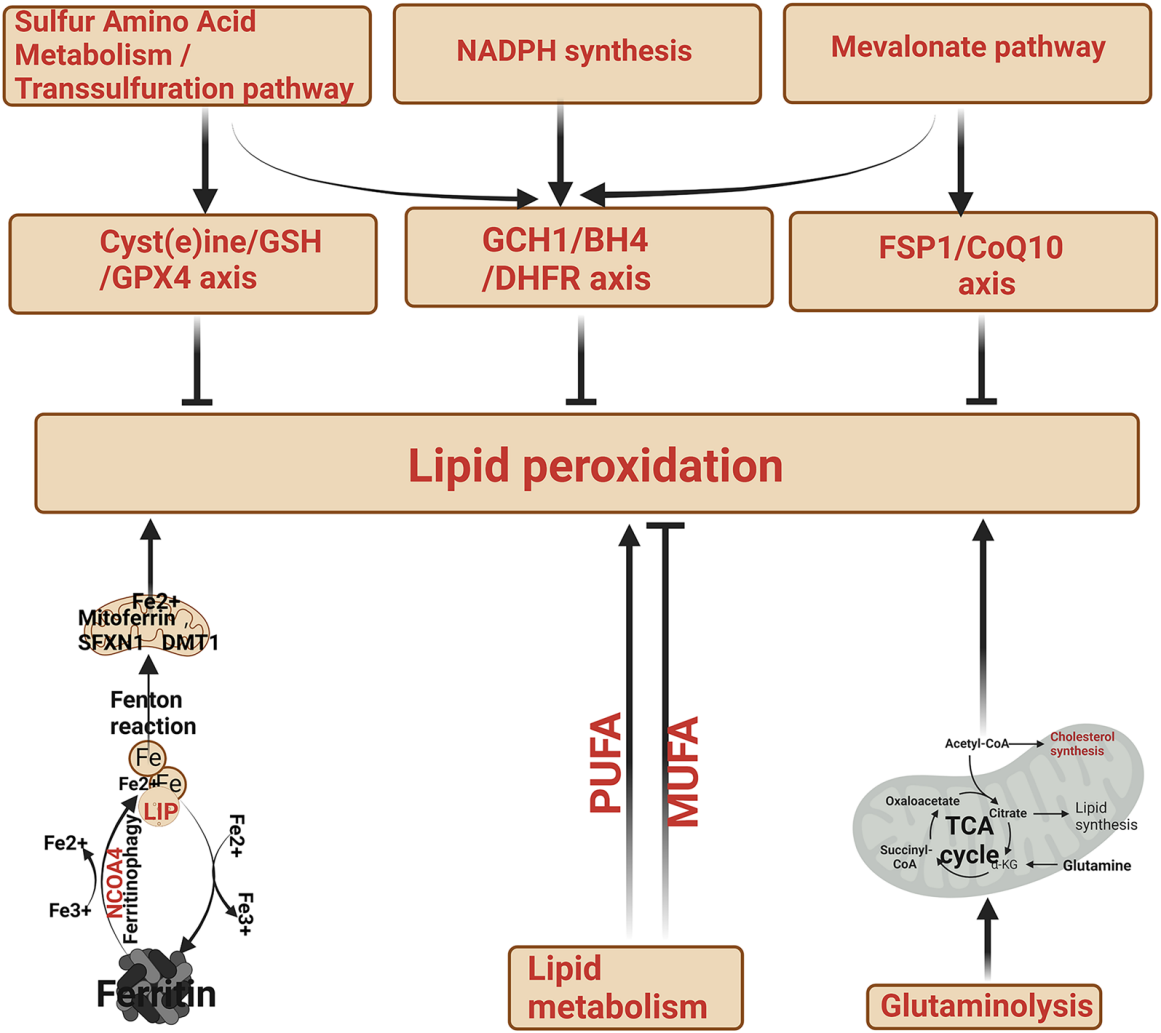
Summary of the relationship between ferroptosis core pathways (*BH4* tetrahydrobiopterin, *GCH1* recombinant GTP cyclohydrolase 1, *GPX4* glutathione peroxidase 4, *DHFR* dihydrofolate reductase, *FSP1* ferroptosis-suppressor protein-1)

The relationship between ferroptosis core pathways is summarized in Fig. 2.

## Disorders of iron metabolism leading to HF

**Fig. 3:**
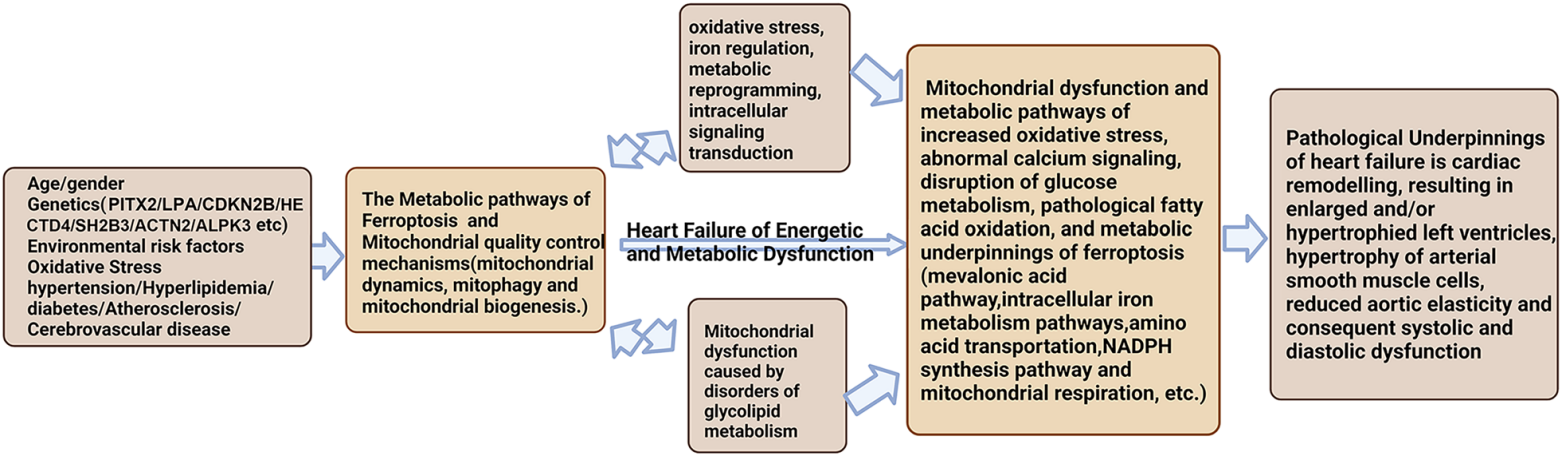
Overview of the relationship between iron metabolism abnormalities and HF

HF is often accompanied by a disordered iron metabolism, which in turn can affect the progression and prognosis of cardiovascular disease. In recent years, the role of iron metabolism in the development of HF has attracted attention from researchers. Here is an overview of the relationship between iron metabolism abnormalities and HF (Fig. 3).

### HF is often accompanied by iron deficiency

Studies have shown that approximately 50% of patients with chronic HF (CHF) experience iron deficiency, which further increases the risk of CHF and mortality. There are three mechanisms underlying iron deficiency in CHF: 1) dietary reduction, gastrointestinal edema, and other factors leading to insufficient iron intake; 2) gastrointestinal bleeding caused by the use of antiplatelet aggregation and antiplatelet drugs, leading to increased iron loss; and 3) increased iron regulatory protein (IRP) expression in CHF patients, causing the body to not release enough iron to meet the needs of the tissue [76]. The first two are called absolute iron deficiency, and the third is functional iron deficiency. These three mechanisms can exist alone or simultaneously. The dysfunction of mitochondrial DNA repair caused by iron deficiency damage the cell's energy supply and heart function. Melenovsky et al. found that the iron content in the left ventricle myocardium of human CHF patients was decreased, the activity of xanthine oxidase and malate dehydrogenase was decreased, the expression of ROS scavenger enzymes [such as catalase, glutathione peroxidase, and superoxide dismutase 2 (SOD2)] was decreased, and the energy production and contractile function of cardiomyocyte were decreased, indicating that myocardial iron deficiency may lead to mitochondrial dysfunction [77]. Mice with TfR1 gene deletion died within the second week after birth, with heart enlargement, poor heart function, mitochondrial respiratory failure, and mitochondrial autophagy inhibition [78].

### HF via ferroptosis during iron overload

Iron overload can be classified into two types: primary hemochromatosis (mutations in genes responsible for iron absorption and regulation), and secondary iron overload (therapeutic interventions, such as repeated blood transfusions to treat hemolytic anemia and excessive iron supplementation to stimulate erythrocyte production in dialysis patients). When the total amount of iron in the body is excessive, the amount of unstable iron pools in the cell increases, and ferritin is deposited in cardiomyocyte cells, leading to HF [79]. In 2012, Dixon et al. named this type of iron-dependent cell death lipid peroxidation-induced ferroptosis [80]. As a novel regulatory cell death mechanism, ferroptosis has become a new focus of research in the field of cardiovascular disease. Understanding the mechanisms by which ferroptosis occurs and develops in HF is a prerequisite for reducing the incidence and mortality of the disease. Iron death can be divided into two stages: the first stage is intracellular iron overload, which generates a large amount of ROS through the Fenton reaction, and the second stage is an imbalance in the intracellular antioxidant system. Currently, research on ferroptosis is mainly focused on four aspects: 1) the System Xc-/glutathione (GSH)/glutathione peroxidase 4 (GPX4) axis is considered the main route involved in ferroptosis. System Xc- is a transaminase located on the surface of the cell membrane that catalyzes the transfer of amino groups, forming a dimer composed of two subunits SLC7A11 and SLC3A2. It can transport cysteine into the cell in a 1:1 ratio and export glutamate outside the cell [81]. GPX4 can remove phospholipid peroxides and prevent ferroptosis. GSH is an effective cofactor of GPX4, and GSH depletion can cause ferroptosis by increasing lipid ROS [82]. Inhibiting the activity of System Xc- will interfere with the uptake of cysteine into the cell, ultimately reducing GSH synthesis and decreasing the activity of GPX4, leading to ferroptosis; 2) the ferroptosis-inhibiting protein 1 (FSP1, also known as AIFM2)-ubiquinone (CoQ10) pathway [83, 84]. Researchers have shown that in some cancer cell lines, even if there is no core anti-ferroptosis system composed of GPX4, the cell can resist ferroptosis. The authors identified FSP1 by genetic sequencing, which must be recruited to the lipid membrane after myristoylation to exert its reductase function [84]. The lipid membrane is rich in CoQ10, and its reduced form CoQH2 acts as an antioxidant to eliminate lipid peroxides [83]. The activity of FSP1 requires the support of NADPH cofactor, so FSP1-CoQ10-NADPH forms an independent anti-ferroptosis system; 3) the GTP cyclohydrolase 1 (GTPCH1)-tetrahydrobiopterin (BH4) pathway [85]. BH4 is an accessory factor involved in the production of aromatic amino acids and nitric oxide. GCH1 is the rate-limiting enzyme in BH4 synthesis. Using a CRISPR-mediated whole-genome activation screen, Kraft et al. found that GCH1 is the most prominent gene involved in suppressing ferroptosis [86]. Upregulation or downregulation of GCH1 controls the endogenous production of antioxidants BH4, leading to the resistance or sensitivity of cancer cells to ferroptosis. Iron protein ferritin h (FtH) is a gene encoding the heavy chain of ferritin, which is specifically downregulated in cardiomyocytes after the induction of cardiomyopathy by doxorubicin. Fang et al. [87] found that the mitochondria in mice with doxorubicin-induced myocarditis accumulate iron and undergo lipid peroxidation, leading to changes in mitochondrial morphology, depolarization of the mitochondrial membrane, and reduction in ATP production. In follow-up studies, the team discovered that the expression of the functional subunit of the cysteine/glutamate transporter SLC7A11 on the cardiomyocyte membrane is downregulated, leading to a lack of cysteine and GSH in the cell, which then triggers cardiomyocyte ferroptosis and mitochondrial failure [88]. SLC7A11/xCT inhibition prevents cardiomyocyte hypertrophy. The NCOA4-mediated autophagy process can selectively degrade iron protein ferritin, leading to an increase in free iron levels in the cell and the induction of ferroptosis [89]. In cells lacking GPX4, DHODH expression is upregulated, leading to mitochondrial lipid peroxidation. DHODH also promotes the regeneration of CoQ10, which captures lipid peroxides on the mitochondrial membrane [90]. Studies have shown that Herceptin can trigger ferroptosis and mitochondrial dysfunction in H9c2 cells [91]. TRIM21 deficiency alleviates the heart toxicity caused by the chemotherapy drug doxorubicin [92].

### HIFs activity is related to iron metabolism, which affects the coding regulation of HF-related genes

Iron is an accessory factor of the prolyl hydroxylase domain (PHD) enzyme, which is involved in the degradation of hypoxia-inducible factors (HIFs). As a result, the accumulation of HIFs and their related metabolic effects are often associated with iron deficiency. HIFs are α/β heterodimer transcription factors that mediate various cellular and systemic responses to changes in oxygen availability in the body [93]. They regulate the transcription of genes involved in angiogenesis, energy metabolism, cell apoptosis, inflammation, and fibrosis [94]. When the heart is subjected to increased load, it undergoes compensatory hypertrophy, which is dependent on heart vessel growth. Chronic heart hypertrophy can lead to HF. Studies have shown that the heart promotes heart vessel growth through HIF-1-dependent induction of angiogenesis factors, but sustained pressure overload leads to the accumulation of p53, which inhibits HIF-1 activity, resulting in damage to heart vessel growth and contractility, leading to HF [95]. Studies have shown that iron depletion at the cellular level inhibits the activity of PHD, leading to nuclear accumulation of HIF-1α, but the impact of this on mitochondrial function remains to be further studied [96]. PHD inhibitors upregulate iron regulatory proteins to improve iron absorption and increase the production of endogenous erythropoietin and enzymes involved in iron metabolism, thereby improving iron utilization [97]. Another study showed that the gene encoding DMT1 is regulated by HIF-2α [97]. Furthermore, studies have used iron chelators to simulate hypoxia to investigate the relationship between HIFs and diseases [98, 99]. It has been found that overexpression of Nr2f2 increases the expression of PGC-1α signals in mice with HF induced by diabetes, leading to exacerbated ferroptosis and mitochondrial dysfunction. Studies have found that Nr2f2 overexpression can aggravate ferroptosis and mitochondrial dysfunction by modulating PGC-1α signaling in mice with diabetes-induced HF. Digoxin protects against ferroptosis in doxorubicin-induced cardiomyopathy rats by modulating HMGB1.

### Calcium channels also become the way for cardiomyocytes to absorb iron

Despite the development of new iron chelators, iron overload still affects patient survival. Understanding the alternative pathways by which iron enters cells is beneficial for the study of new treatments for iron overload diseases. Only iron bound to transferrin can enter cardiomyocyte cells through TfR1. Non-transferrin-bound iron (NTBI) is transported into cardiomyocyte cells mainly through two transporters: DMT1 and L-type Ca2 + channels (LTCC). However, the expression of DMT1 mRNA in cardiomyocyte cells has been shown to decrease with increasing levels of intracellular iron in adult human hearts [100]. In 1999, Tsushima et al. first discovered that cardiomyocyte iron uptake is through L-type Ca2 + channels, and blocking this channel may be helpful in treating patients with high serum iron levels [101]. Overexpression of cardiomyocyte-specific LTCC leads to increased iron accumulation and oxidative damage in cardiomyocyte cells, with a proportional increase in Ca2 + current. LTCC blockers (such as amlodipine and verapamil) reduce iron accumulation in cardiomyocyte cells, reduce oxidative stress, and protect against diastolic and systolic dysfunction [102]. NTBI entering cardiomyocyte cells is rapidly oxidized and reduced to trivalent iron, which is then trapped in the cytoplasm and converted to insoluble ferric hemoglobin or reactive unstable iron pools causing oxidative damage via iron-mediated oxidation. Increases in cytoplasmic iron also lead to iron uptake into mitochondria, which are integrated into the Fe–S clusters and used for erythropoiesis [103]. The stability of iron in the mitochondria requires frataxin, which provides critical antioxidant defense against iron-dependent radicals in the mitochondria [104]. Another potential pathway involving iron absorption in the heart is the T-type Ca2 + channel (TTCC). Under physiological conditions, TTCC expression in the heart disappears after birth [105]. However, it can reappear in the heart under certain pathological conditions, including myocardial infarction, HF, and iron overload cardiomyopathy [106]. Research has shown that Fe2 + can inhibit Ca2 + current and can enter cardiomyocyte cells through TTCC under iron overload conditions [107]. Treatment with TTCC blockers (such as ethinylestradiol) can significantly reduce iron absorption in cultured cardiomyocyte cells and iron overload models in vivo, improving heart function [108]. However, further research is needed in clinical and in vivo environments to confirm the role of these channels in heart iron absorption and their potential application in preventing iron overload cardiomyopathy.

### Regulation of NRF2-ferroptosis pathway on HF

Nrf2 is a transcriptional factor that is widely distributed throughout the body. In the process of ferroptosis, almost all genes that regulate ferroptosis are related to Nrf2 regulation, which mainly includes genes that regulate glutathione (GSH) synthesis, system xc–induced cysteine supply, glutathione reductase, and GPX4, the activity of which is crucial for the activity of GPX4. NADPH regeneration, including glucose-6-phosphate dehydrogenase, phosphoglycerate dehydrogenase, and malic enzyme, and iron regulation, including iron excretion and storage, heme synthesis (degradation), and ferritin synthesis [109, 110]. Nrf2 physically binds to the mitochondria and can track and respond to changes in mitochondrial function. It also interacts with peroxisome proliferator-activated receptor gamma coactivator 1 (PGC-1), which regulates mitochondrial biogenesis and autophagy [111]. Silence of the mouse Nr2 gene has been shown to damage mitochondrial function, while activation of the Nrt2 gene improves mitochondrial function and resistance to stress [112]. Therefore, Nrf2 is closely involved in the process of ferroptosis and is an important regulator of the oxidative and anti-oxidative balance. Normally, Nrt2 binds to the negative regulator Kelch-like ECH-associated protein 1 (Keap1) in the cytosol, and Keap1 continuously phosphorylates and degrades Nrf2 through ubiquitination and proteasome degradation. This maintains low levels of Nrf2 signaling. In states of increased oxidative stress, Nrf2 and Keap1 dissociation and Nrf2 nuclear translocation are accelerated [113]. In the nucleus, Nrf2 interacts with the antioxidant response element (ARE) in the promoter region of target genes and transcribes them. Nrf2 thus participates in the regulation of cardiovascular disease [114, 115]. A study has shown that mice with knockout of the Nrl2 gene develop faster HF, with higher levels of atrial natriuretic factor (ANF) and B-type natriuretic peptide (BNP), indicating that Nrf2 protects cardiomyocytes from damage [116]. Literature has reported that overexpression of Nrf2 can reduce ROS production, myocardial hypertrophy, and myocardial cell fibrosis in a mouse model of aortocaval compression, indicating that Nrf2 plays an important role in cardiovascular physiological changes [117, 118]. The regulatory mechanism of the Nrf2-ferroptosis pathway in HF is mainly mediated by Hmox1, a heart-protective protein, but overexpression of which can lead to HF. Administration of doxorubicin-induced HF can be activated by Nrf2, transported to the nucleus, and combined with the ARE to activate transcription, leading to the upregulation of Hmox1, the release of free iron, and the accumulation of lipid peroxides in the mitochondria, resulting in ferroptosis and ultimately leading to HF. Studies have shown that the model of ferroptosis induced by doxorubicin is mediated by Hmox1, and the expression of Hmox1 is regulated by multiple transcriptional factors, such as Nrf2, AP-1, and YY1. However, only the Nrf2-mediated regulatory process exhibits resistance to ferroptosis [119]. Therefore, suppressing ferroptosis or controlling the upregulation of Hmox1 is conducive to alleviate doxorubicin-induced HF.

### The mechanism of non-coding RNA-mediated ferroptosis involved in HF

circSnx12 targets miR-224-5p to participate in ferroptosis during HF [120]. The TLR4/NADPH oxidase 4 pathway plays a role in promoting cell death through autophagy and ferroptosis during HF [121]. OMA1-mediated integrated stress response can prevent ferroptosis in mitochondrial cardiomyopathy [122]. Inhibition of long noncoding RNA ZFAS1 via sponge miR-150-5p weakens ferroptosis and activates CCND2 against diabetic cardiomyopathy [123]. miR-375-3p regulates ferroptosis via GPX4-mediated pathway to promote heart fibrosis [124]. The neutrophil-like cell membrane-encapsulated long noncoding RNA AABR07017145.1 functions as a therapeutic agent for heart hypertrophy by suppressing ferroptosis through blocking CMEC [125].

Iron overload caused by endoplasmic reticulum heme degradation triggers ferroptosis in myocardial ischemia–reperfusion injury [126]. Zheng et al. found that thoracic aorta constriction (TAC) can cause ferroptosis in mouse cardiomyocyte and induce HF. CircSnx12 and FTH1 expression were downregulated in the heart tissue of HF mice, while miR-224-5p expression was upregulated. These findings suggest that the expression of circSnx12, miR-224-5p, and FTH1 is related to HF. circSnx12 overexpression can protect cardiomyocytes and reduce their sensitivity to ferroptosis. In contrast, miR-224-5p overexpression can antagonize the protective effect of circSnx12 overexpression on cardiomyocyte ferroptosis. circSnx12 maintains the intracellular iron metabolism homeostasis by competing with FTH1 for miR-224-5p binding, and regulates ferroptosis in mouse cardiomyocyte of HF [127].

## Treatment strategies

While studying the mechanisms of iron metabolism and cardiovascular disease, therapeutic strategies that adjust iron metabolism and improve myocardial iron content are also being actively studied. Targeting iron dysmetabolism and its pathogenic processes may become a new therapeutic strategy for treating HF.

### Iron deficiency treatment strategy

Functional iron deficiency and absolute iron deficiency can coexist, and functional iron deficiency can promote absolute iron deficiency by continuously damaging iron intake [128]. Absolute iron deficiency treatment focuses on improving iron storage, improving anemia, and optimizing iron absorption, while functional iron deficiency treatment focuses on controlling potential diseases.

#### Oral iron supplementation

A statistical study has shown that iron supplementation can reduce the hospitalization rate of patients with HF, enhance heart function, and improve quality of life [129]. Oral iron supplements commonly used include ferrous salts (such as ferrous sulfate) and dextran iron, lactoferrin, and ferritin complex, etc. However, the use of ferrous salts for treatment may be influenced by gastrointestinal adverse reactions [130]. A new oral therapy that combines ferric ions with carriers to optimize absorption while reducing gastrointestinal side effects is currently being studied. In a Phase III clinical expansion study, mefenamic acid–ferric hydroxide was effective and well tolerated in patients with iron deficiency anemia associated with inflammatory bowel disease [131]. Research has shown that a new type of nanoparticle iron supplement (dietary ferritin-like particles of ferric hydroxide and hexanoic acid succinic acid iron) can be safer and more effective than ferrous sulfate as an oral iron supplement and has the potential to treat iron deficiency anemia in humans [132].

#### Intravenous iron administration

When oral iron supplements are not tolerated, ineffective, cannot be used, or require rapid supplementation of depleted iron stores, intravenous iron supplementation is required [133]. Compared to oral supplementation, intravenous supplementation is faster and more effective, requiring fewer doses, and has less gastrointestinal side effects, and absorption is not affected by inflammation or other factors [134]. Carboxylated maltose iron is the preferred formulation because it seems to increase blood hemoglobin parameters more rapidly and effectively [135]. Lopez-Vilella et al. conducted a study on 565 outpatients with diagnoses of chronic HF for more than 5 years, and found that intravenous administration of carboxylated maltose iron improved the ejection fraction and clinical status of patients with iron deficiency and chronic HF [136].

#### Reduce hepcidin

As mentioned earlier, when there is an iron deficiency, the secretion of hepcidin decreases, leading to increased cell iron excretion and reduced plasma iron levels. Studies have shown that vitamin D [137] and heparin [138] can inhibit the production of hepcidin, providing potential value for the treatment of iron deficiency. With further research on the hepcidin signaling pathway, targeted inhibitors of soluble hemojuvelin (HJV) and bone morphogenetic protein receptors (BMPR) as well as inhibitors of IL-6/JAK/STAT signaling have become therapeutic options for treating iron deficiency [139].

### Treatment strategies for iron overload

Iron overload leads to saturation of transferrin and ferritin, increasing non-transferrin-bound iron (NTBI), which accelerates iron deposition in tissues, especially in excitable tissues containing high levels of Ca2 + channels. High levels of Ca2 + channels in the heart tissue will transmit Fe2 + ions into the cell through oxidative–reductive reactions, producing excessive free radicals that disrupt the cell's oxidative–reductive balance and cause oxidative stress. Free radicals then combine with various cell components to produce toxicity in the heart [140, 141]. NTBI can also directly activate fibroblasts and promote their proliferation and differentiation into myofibroblasts, leading to increased fibrosis in heart tissue [142].

#### Phlebotomy and iron chelators

Treatment of iron overload requires reducing systemic iron levels or preventing iron from entering tissues. Blood transfusion and iron chelators are two common methods for reducing systemic iron [143]. When hemoglobin is within the normal range (no anemia) and erythrocyte production is not affected, blood transfusion is used. This method is mainly used to treat primary hemochromatosis. When patients with iron overload have anemia, iron chelators are used to remove excessive iron from the body. Continuous development of orally bioavailable drugs with good long-term efficacy and safety has become a goal for the treatment of iron overload. In addition, different treatment strategies may be used depending on the mode of action.

#### Inhibition of ferroptosis

Research has shown that ferroptosis inhibitors, iron chelators, mitochondrial reductases, HO-1 inhibitors, and low-iron diets can effectively prevent and treat ferroptosis-mediated HF (HF) [79]. GSH, ferrostatin-1, liproxstatin-1, ascorbic acid (AsA), vitamin E, and CoQ10 are lipid antioxidants that inhibit ferroptosis. Specifically, a mitochondrial antioxidant (Mito-TEMPO) can effectively prevent ferroptosis and protect heart function. Most iron chelators mainly bind Fe2 + outside the cell, while right isomerase and ciclopirox (CPX) can bind Fe2 + inside the cell. Acyl-CoA synthetase long-chain family member 4 (ACSL4) can convert arachidonic acid and aldosterone into arachidonic acid CoA and aldosterone CoA, respectively, and participate in the synthesis of negatively charged membrane phospholipids. However, long-chain polyunsaturated fatty acids (PUFAs) on the membrane are often oxidized, especially under the induction of factors such as RSL3, leading to cell ferroptosis [53]. Rosiglitazone and pioglitazone can protect cells from ferroptosis by inhibiting the activation of long-chain PUFAs mediated by ACSL4 [144]. Additionally, kalirigrigin can alleviate ferroptosis in mice with preserved ejection fraction and improve HF [145]. The SGLT-2 inhibitor engegridin improves myocardial function and fibrosis in non-diabetic mice treated with adriamycin, and reduces proinflammatory cytokines [146]. Activating the Nrf2/HO-1 signaling axis has also been shown to inhibit ferroptosis and prevent lung ischemia–reperfusion injury [147], but its application in HF still needs further study. Treatment with atorvastatin improves heart function and remodeling by reducing ferroptosis in isoproterenol-induced HF [148]. LV16A, a protein kinase 43 (PK43) agonist, reverses LV dysfunction and ferroptosis in HF caused by myocardial infarction, preserving ejection fraction [149]. Edaravone reduces ferroptosis by inhibiting excessive autophagy following myocardial infarction, preserving heart function [150].

#### Antioxidants

Iron overload leads to an increase in ROS production, which not only damages cell components, but also serves as a potential basis for the development of diseases [151]. Many studies have shown that antioxidants can reduce heart oxidative stress, reduce heart iron deposition, and improve heart function induced by iron overload [152, 153]. A clinical trial conducted for 3 months showed that oral N-acetyl-L-cysteine (NAC), a thiol antioxidant, can be used as a complementary therapy for patients with HF [89]. Mice with knockout of the HJV gene showed increased heart iron deposition and mortality, as well as increased oxidative stress and myocardial fibrosis, leading to late-stage iron overload cardiomyopathy. Treatment with resveratrol could suppress the iron-mediated oxidative stress and myocardial fibrosis, while activating the p-Akt and p-AMPK signaling pathways [154]. In addition to antioxidant monotherapy, the combination of antioxidants and iron chelators can enhance therapeutic effects by reducing tissue iron accumulation and reducing oxidative stress under conditions of iron overload, improving organ function [155, 156].

#### Calcium channel blockers

Iron ions can also enter the heart through Ca2 + channels. Therefore, calcium channel blockers (such as amlodipine and verapamil) can be used to reduce iron accumulation in the heart and prevent iron overload-induced HF. Calcium channel blockers can promote myocardial microvascular perfusion by dilating coronary arteries and improving coronary endothelial function. Calcium channel blockers such as amlodipine also have antioxidant properties, which can help counteract the oxidative effects of iron overload [157]. When used in combination with iron chelators, amlodipine is more effective in reducing cardiac iron overload than iron chelators alone [158]. Verapamil can reduce the increase in serum ferritin and cardiac iron deposition induced by ferrous sulfate, improve oxidative stress, and protect against the cardiac functional and structural changes caused by iron overload [159]. Mitochondrial calcium uniporter (MCU) blockers exert a protective effect by preventing ROS generation, mitochondrial depolarization, and reducing mitochondrial swelling caused by iron overload, which may be an effective therapy for preventing cardiac mitochondrial dysfunction caused by iron overload [160].

#### Increase hepcidin

During iron overload, hepcidin secretion increases, leading to decreased cellular iron efflux and lower plasma iron levels. Therefore, hepcidin mimetics may be a potential therapeutic approach [161]. Studies have shown that this approach is more suitable for maintenance therapy, as these drugs do not reduce iron stores in the liver. Increasing hepcidin production by increasing positive regulators (such as BMP6) or inhibiting negative regulators of hepcidin signaling is also a therapeutic strategy for iron overload [162].

#### Tissue inhibitors of metalloproteinases

Tissue inhibitors of metalloproteinases (TIMPs) can also mediate cardiac remodeling, hypertrophy, and fibrosis in heart disease. Studies have shown that TIMP3 is a key regulator of iron-mediated cardiac injury, and mice lacking TIMP3 exhibit severe cardiac structural changes and functional impairment after iron overload treatment [163]. Therefore, exploring the mechanisms of iron metabolism and developing new drugs targeting iron metabolism are of great significance for the treatment of heart disease.

## Molecular mechanism of natural products regulating ferroptosis and improving HF

### Natural compounds

#### Berberine

Berberine (BBR) is an isoquinoline alkaloid isolated from the *Coptidis Rhizoma*. It has been found to possess various pharmacological effects, including anti-infective, anti-diabetic, anti-aging, anti-cancer, antioxidant, and cardioprotective properties [164, 165]. BBR pretreatment can reduce myocardial infarction (MI) volume and improve heart function by inhibiting cardiac fibrosis, inflammatory response, cardiomyocyte apoptosis, and oxidative stress damage [166, 167]. Yang et al. found that in H9c2 cardiomyocytes, BBR reduced erastin and RSL3-induced loss of cell viability. Additionally, BBR reduced ROS accumulation and lipid peroxidation in iron-overloaded cells. Furthermore, quantitative polymerase chain reaction results showed that Ptgs2 mRNA decreased in BBR-treated cells. In rat neonatal cardiomyocytes, BBR also reduced RSL3-induced loss of cell viability. These results suggest that BBR inhibits iron toxicity by reducing ROS production and lipid peroxidation in iron-treated cardiac cells [168].

#### Resveratrol

Resveratrol is a non-flavonoid polyphenol organic compound with various pharmacological effects, such as antioxidant, anti-inflammatory, anticancer, and cardiovascular protective activities [169, 170]. Studies have shown that resveratrol can attenuate oxidative stress damage, reduce Fe2 + levels, and inhibit ferroptosis induced by oxygen glucose deprivation/reoxygenation (OGD/R) in an I/R model of H9c2 cells [169]. In a myocardial I/R model of diabetic rats, I/R activates ubiquitin-specific protease 19 (USP19) and Beclin1, leading to ferroptosis, while resveratrol can inhibit iron death by downregulating USP19/Beclin1 and upregulating GPX4 and FTH1, thus reducing myocardial cell I/R injury. A recent analysis showed that resveratrol can alleviate myocardial damage by inducing KAT5/GPX4 and inhibiting iron death in myocardial infarction [171]. Zeng et al. found that resveratrol can protect against MI/R injury by reducing oxidative stress and attenuating ferroptosis, suggesting its potential as a preventive drug for MI/R injury [172].

#### Baicalin

Baicalin is a flavonoid derivative isolated from the traditional Chinese medicine Scutellaria baicalensis Georgi, with significant pharmacological activities, including anti-inflammatory, immunomodulatory, and antioxidant effects [173, 174]. Baicalin improves cardiac function and myocardial fibrosis in rats with myocardial infarction, possibly through the p38 phosphorylation and TGFβ1/Smad2 pathway, exhibiting a protective effect against MI/R injury [175]. Fan et al. found that baicalin improves ST-segment elevation on electrocardiogram, coronary blood flow, left ventricular systolic pressure, infarct area, and pathological changes in rats with MI/R injury, and inhibits the decrease of cell viability induced by OGD/R in H9c2 cells. Lipid peroxidation, iron overload, TfR1 activation, and nuclear receptor coactivator factor 4-mediated iron autophagy, enhanced in both in vivo and in vitro models, can be reversed by baicalin treatment. In addition, overexpression of ACSL4 weakens the protective effect of baicalin on H9c2 cells. These results indicate that baicalin regulates ferroptosis by inhibiting ACSL4, preventing MI/R injury, and providing a new potential target for preventing MI/R injury [176].

#### Cyanidin-3-O-glucoside

Cyanidin-3-O-glucoside (C3G), belonging to anthocyanins, is a flavonoid widely distributed in plants, especially in black rice, black bean, and purple sweet potato [177, 178]. C3G is one of the main components of mulberry anthocyanins, which have been widely used in food and health products. Anthocyanins have various therapeutic effects, such as antioxidation, anti-atherosclerosis, anti-insulin resistance, and regulation of blood lipids, among which C3G is the main active ingredient [179, 180]. Studies have shown that C3G can reduce myocardial infarct size, alleviate pathological changes, inhibit ST-segment elevation, and reduce the expression of proteins related to oxidative stress and iron death in the rat model of myocardial ischemia–reperfusion (I/R). C3G can also inhibit the expression of USP19, Beclin1, nuclear receptor coactivator 4, and microtubule-associated protein 1 light chain 3II/LC3I. In addition, in H9c2 cells induced by OGD/R, C3G can reduce oxidative stress, downregulate LC3II/LC3I, decrease the number of autophagosomes, downregulate TfR1 expression, upregulate FTH1 and GPX4 expression, and promote K11 ubiquitination of Beclin1. Therefore, C3G can reduce MI/R injury by inhibiting ferroptosis in vivo and in vitro according to the results of this study [181].

#### Naringenin

Naringenin is a natural flavonoid compound with multiple pharmacological effects, including antibacterial, anti-inflammatory, antioxidant, anticancer, anti-tumor, and anti-atherosclerosis activities. Studies have shown that naringenin inhibits ferroptosis in MI/R injury [182, 183]. It exerts cardioprotective effects by alleviating pathological damage, inflammation, and lipid peroxidation induced by I/R in rat myocardial tissue. Naringenin activates the Nrf2/SLC7A11/GPX4 axis by upregulating Nrf2, SLC7A11, GPX4, FTH1, and ferroportin-1, and downregulating NOX1 NADPH oxidase to inhibit ferroptosis. At the cellular level, the ferroptosis inducer erastin can counteract the protective effect of naringenin on I/R-induced H9c2 cardiomyocytes [184].

#### Gossypol acetic acid

Gossypol acetic acid (GAA) is a natural product extracted from cottonseed, which can inhibit oxidative stress damage [185, 186]. Studies have shown that GAA can attenuate MI/R injury by inhibiting ferroptosis in OGD/R-mediated H9c2 cells; in H9c2 and rat cardiomyocytes treated with ferroptosis inducers erastin, RSL3, and Fe-SP, GAA can protect H9c2 cells from ferroptosis induction by reducing the production of malondialdehyde and ROS, chelating iron content, and downregulating Ptgs2 mRNA levels. GAA can also prevent cardiomyocyte death and lipid peroxidation induced by OGD/R. In isolated rat hearts, GAA can significantly reduce infarct size, decrease lipid peroxidation, lower PTGS2 and ACSL4 mRNA levels, decrease ACSL4 and Nrf2 protein levels, and upregulate GPX4 protein levels. Therefore, GAA may play a cytoprotective role in ferroptosis-induced cardiomyocyte death and MI/R injury [187].

#### Astragaloside IV

Astragaloside IV (AS-IV) is one of the main active components of Astragalus membranaceus, a widely used traditional Chinese herb [188, 189]. AS-IV has been reported to possess multiple pharmacological effects, such as protecting the heart, antioxidation, anti-inflammation, anti-tumor, anti-apoptosis, and regulating blood glucose levels. AS-IV has therapeutic effects on various cardiovascular diseases, including improving myocardial fibrosis, inhibiting inflammatory reactions, anti-oxidative stress, regulating myocardial cell energy metabolism, enhancing myocardial contractility, and preventing myocardial cell apoptosis [190, 191]. Tian et al. found that AS-IV can reverse doxorubicin-induced myocardial injury in rats, possibly by activating the Nrf2/GPX4 pathway to alleviate myocardial ferroptosis induced by DOX [192].

#### Ophiopogonin D (OPD)

OPD is a bioactive steroidal glycoside extracted from Ophiopogon japonicus, which has pharmacological effects such as anti-inflammatory, antioxidant, cough suppressant, and anti-thrombotic activities [193, 194]. In terms of HF, OPD significantly improves myocardial injury caused by hypoxia, and its mechanism may be related to regulating GSH metabolism, inhibiting myocardial oxidative stress, and endoplasmic reticulum stress levels [195]. Lin et al. found that OPD could treat ISO-induced rat HF and induce changes in CYP2J3 expression in cardiac tissue. At the cellular level, OPD can induce the expression of CYP2J2 and CYP2J3, and this induction is mediated by PXR. In addition, it was found that the two isomers of OPD, OPD and OPD', have different pharmacological effects on H9c2 cells. OPD can reverse OPD'-induced ferroptosis, thereby protecting myocardial cells [196].

#### Artesunate

Artemether is the preferred drug for treating complicated malaria, with various biological activities including anti-inflammatory, immune modulation, anti-fibrotic, and anti-lipid peroxidation effects [197]. It can reduce hypoxia/reoxygenation-induced H9c2 cell apoptosis and myocardial ischemia/reperfusion injury through anti-inflammatory, anti-apoptotic, and antioxidant effects [198], and significantly inhibit endoplasmic reticulum stress by blocking the PERK/eIF2α/ATF4/CHOP pathway, thereby alleviating ulcerative colitis-induced intestinal barrier inflammation and pathological damage [199]. Hong et al. found that artemether could inhibit OGD/R-induced inflammation, iron accumulation, and lipid peroxidation in myocardial cells, alleviate ferroptosis, enhance cell viability, and relieve cell damage by inhibiting the PERK/ATF4/CHOP pathway activation [200].

#### Luteolin

Luteolin is an important flavonoid compound found in various natural plants, with multiple pharmacological activities such as anticancer, antioxidant, antibacterial, and anti-inflammatory effects [201, 202]. Luteolin may exert cardioprotective effects in ischemia/reperfusion (IR) by inhibiting oxidative damage, cell apoptosis, and autophagy, possibly through the activation of the AKT/mTOR/STAT3 signaling pathway [203]. Ma et al. found that luteolin can inhibit oxidative stress and attenuate Ang II-induced cardiomyocyte hypertrophy, possibly by enhancing Nrf2 nuclear transcription and activating the Nrf2/Gpx4 pathway, thus inhibiting ferroptosis [204].

#### Puerarin

Puerarin is one of the main monomers of total flavonoids from Pueraria lobata, and is an isoflavone phytoestrogen with important pharmacological effects, minimal adverse reactions, and high safety [205, 206]. It has been reported that puerarin exhibits various pharmacological activities, including anti-inflammatory, anti-oxidative stress, anti-apoptosis, and anti-autophagy effects, which are beneficial for alleviating myocardial ischemia/reperfusion injury and treating ischemic cardiovascular diseases [207, 208]. Jiang et al. found that puerarin could inhibit sorafenib-induced myocardial ferroptosis, and has a good protective effect against the cardiotoxicity of sorafenib. β-Carotene can antagonize the cell-protective and lipid peroxidation-inhibiting effects of puerarin, and the main molecular mechanism underlying the inhibition of endoplasmic reticulum stress by puerarin may be through scavenging lipid ROS [209]. Liu et al. found that puerarin can prevent pressure overload-induced HF by alleviating ferroptosis [210].

#### Schizandrin B

Schisandrae Chinensis Fructus has the functions of astringency, thirst-quenching, kidney-nourishing, and tranquilizing [211]. It has a complex chemical composition and exhibits good pharmacological effects such as anti-oxidation, anti-tumor, liver protection, and anti-inflammation. Schizandrin B, a component of Schisandrae Chinensis Fructus, has been found to possess anti-inflammatory, anti-oxidative, and anti-tumor properties [212, 213]. Currently, it has been widely used in the treatment of liver disease, tumors, cardiovascular disease, sepsis, and neurological disorders, showing remarkable therapeutic effects against inflammation-related diseases such as rheumatoid arthritis, pneumonia, cardiovascular disease, and sepsis [214, 215]. Yang et al. found that Schizandrin B can inhibit ferroptosis and alleviate myocardial injury in diabetic mice, and its mechanism of action may be related to the activation of the Nrf2/HO-1/GPX4 signaling pathway [216].

#### Salvianolic acid B

Salvianolic acid B is a highly active component in Danshen water extract, which has anti-oxidative, anti-inflammatory, and anti-fibrotic effects. It has been studied as a potential drug for cardiovascular disease for many years [217, 218]. Studies have found that salvianolic acid B can increase the expression of Cx43 protein in the interventricular septal defect rabbit myocardium [219], and Cx43 can reduce mitochondrial reactive oxygen species production and protect against doxorubicin-induced cardiac injury [220]. Liu et al. found that salvianolic acid B could protect the heart of MI rats by upregulating the expression and phosphorylation of Cx43 protein, improving its distribution in myocardium, and resisting cellular ferroptosis [221].

#### Apigenin

Apigenin is a common natural flavonoid compound, which is abundant in vegetables and fruits commonly consumed in daily life [222, 223]. It mainly involves signaling pathways such as phosphoinositide 3-kinase/protein kinase B (PI3K/AKT), signal transducer and activator of transcription 3 (STAT3), WNT, and mitogen-activated protein kinase/extracellular signal-regulated kinase (MAP/ERK) [224, 225]. Liu et al. found that Apigenin could improve acute myocardial I/R injury by inhibiting ferroptosis and apoptosis. Acute myocardial I/R injury is associated with ferroptosis, and Fer-1 significantly reduces this injury. Apigenin improves ferroptosis-associated acute myocardial I/R injury by activating the AMPK/Nrf2/HO-1 signaling pathway [226].

#### Thymoquinone

Thymoquinone is a natural monomer extracted from the seeds of Nigella sativa, a plant of the Ranunculaceae family, which is low-toxicity and highly efficient, possessing a range of biological activities including anti-tumor, anti-inflammatory, anti-oxidative stress, anti-fibrosis, protection against ischemia–reperfusion injury, antiviral, analgesic, anti-anxiety/convulsant, and radioprotective effects [227, 228]. Luo et al. found that thymoquinone can alleviate DOX-induced cardiotoxicity, which may be achieved by activating the Nrf2/HO-1 signaling pathway to relieve ferroptosis in mouse cardiac cells, thereby mitigating DOX-induced cardiotoxicity [229].

**Fig. 4:**
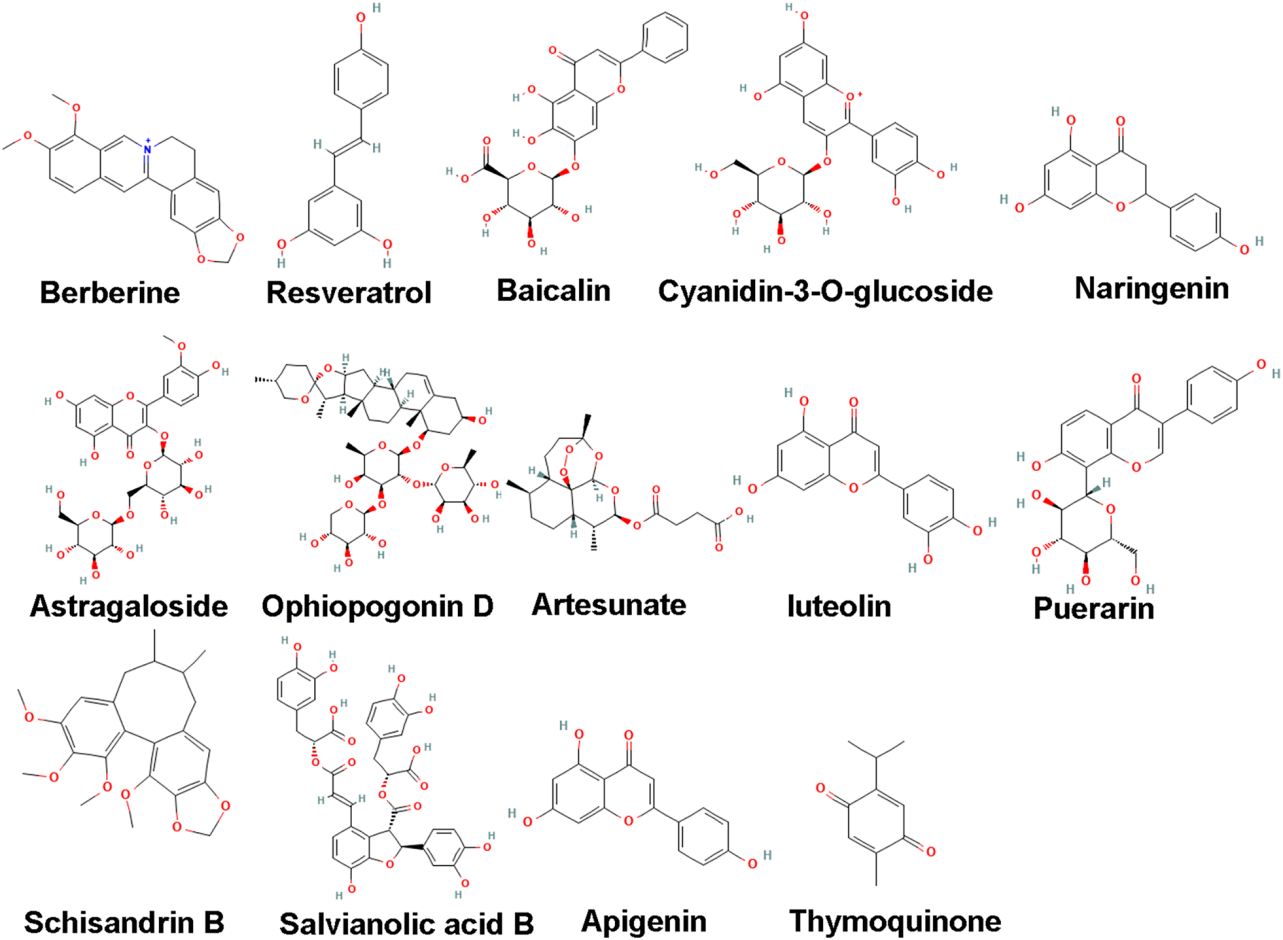
The structures of natural compounds

The mechanism of natural compounds is summarized in Table 1 and the structures of natural compounds are shown in Fig. 4.

**Table 1:** Summary of the mechanism of natural compounds

Natural compounds	Function	Reference
Berberine	Inhibits iron toxicity by reducing ROS production and lipid peroxidation in iron-treated cardiac cells	[168]
Resveratrol	Reduce oxidative stress and attenuating ferroptosis	[172]
Baicalin	Regulate ferroptosis by inhibiting ACSL4	[176]
Cyanidin-3-O-glucoside	Upregulate FTH1 and GPX4 expression	[181]
Naringenin	Activates the Nrf2/SLC7A11/GPX4 axis by upregulating Nrf2, SLC7A11, GPX4, FTH1, and ferroportin-1, and downregulating NOX1 NADPH oxidase to inhibit ferroptosis	[184]
Gossypol acetic acid	Decrease ACSL4 and Nrf2 protein levels, and upregulate GPX4 protein levels	[187]
Astragaloside IV	Activate the Nrf2/GPX4 pathway to alleviate myocardial ferroptosis induced by DOX	[192]
Ophiopogonin D	Regulate ferroptosis, thereby protect myocardial cells	[196]
Artesunate	Inhibit the PERK/ATF4/CHOP pathway activation	[200]
Luteolin	Enhance Nrf2 nuclear transcription and activate the Nrf2/Gpx4 pathway	[204]
Puerarin	Alleviate ferroptosis	[210]
Schizandrin B	Activate the Nrf2/HO-1/GPX4 signaling pathway	[216]
Salvianolic acid B	Upregulate the expression and phosphorylation of Cx43 protein	[221]
Apigenin	Activate the AMPK/Nrf2/HO-1 signaling pathway	[226]
Thymoquinone	Activate the Nrf2/HO-1 signaling pathway	[229]

### Traditional Chinese medicine prescription

#### Qidi Qiangxin capsule (QDQXC)

Wang et al. found that the main mechanisms of QDQXC in treating chronic HF include enhancing myocardial contractility, improving water and sodium retention, inhibiting the overactivation of the neuroendocrine system, regulating myocardial fibrosis, inhibiting apoptosis of myocardial cells, suppressing inflammatory factors, improving myocardial energy metabolism, and protecting endothelial function [230]. Analysis showed that QDQXC can exert its pharmacological effects through multiple pathways and signaling pathways, improving heart function in patients with HF. Liu et al. found that QDQXC can alleviate oxidative damage in H9c2 cardiomyocytes induced by doxorubicin, inhibit the occurrence of ferroptosis, and may reduce doxorubicin-induced ferroptosis in H9c2 cardiomyocytes by upregulating the expression of system xc- and GPX4 through the Nrf2 signaling pathway [231].

#### Yiqi Huoxue Fang (YQHXF)

YQHXF has various pharmacological effects, including scavenging of oxygen free radicals, anti-oxidative stress, anti-atherosclerosis, and cardioprotection [232]. Wang et al. found that YQHXF improved cardiac function, reduced myocardial inflammatory cell infiltration, and alleviated myocardial fibrosis and ventricular remodeling in rats with acute myocardial infarction. This effect may be attributed to the downregulation of AMPK and Beclin1 protein phosphorylation, upregulation of SLC7A11 and GPX4 protein expression, and inhibition of autophagy and ferroptosis after myocardial infarction, leading to inhibition of ventricular remodeling [233].

#### Shexiang Baoxin Wan (SXBXW)

SXBXW has the effects of promoting blood circulation, relieving pain, and clearing the mind and eliminating turbidity [234, 235]. SXBXW can quickly dilate coronary arteries and has good efficacy for patients with stable angina pectoris [235]. In addition, modern research has found that Moschus Heart-Protecting Pill has multiple effects, such as inhibiting myocardial remodeling, improving vascular endothelial function, promoting vascular neogenesis, reducing plaques, and improving myocardial ischemia–reperfusion injury [236, 237]. Ye et al. found that SXBXW can alleviate ferroptosis of myocardial cells by regulating the miR-144-3p/SLC7A11 signaling pathway [238].

#### Lu Hong Fang (LHF)

LHF, as a traditional Chinese medicine formula, has the characteristics of complex composition, diverse action pathways and targets, and is mainly used to treat angina pectoris, asthma, and other related conditions [239]. Previous clinical research has shown that Lu Hong Fang can effectively improve clinical symptoms of HF after myocardial infarction and regulate coronary microcirculation [240]. LHF pretreatment can increase the GSH content in the serum of rats with reperfusion injury [241], and alleviate MIRI. Cai et al. found that LHF can alleviate myocardial ischemia–reperfusion injury, and its mechanism may be related to the upregulation of the SLC7A11/GPX4 pathway, activation of the Keap-1/Nrf2/ARE antioxidant signaling pathway, and inhibition of ferroptosis [242, 243].

#### Yu Xue Bi (YXB)

Research has shown that the main active ingredients of YXB are quercetin, cryptotanshinone, tanshinone IIA, and ferulic acid [244]. YXB has been found to have a protective effect on myocardial cell damage, and through screening of its active ingredients using an H9c2 myocardial cell injury model, the main active ingredients of YXB in protecting against myocardial cell damage were identified. Huang et al. found that YXB may inhibit myocardial cell ferroptosis and improve myocardial hypertrophy by activating the DJ-1/GPX4 signaling pathway, thereby exhibiting anti-HF effects [245].

The mechanism of traditional Chinese medicine prescription is summarized in Table 2.

**Table 2:** Summary of the mechanism of traditional Chinese medicine prescription

Traditional Chinese medicine prescription	Function	Reference
QDQXC	Upregulate the expression of system xc- and GPX4 through the Nrf2 signaling pathway	[231]
YQHXF	Downregulate AMPK and Beclin1 protein phosphorylation, upregulate SLC7A11 and GPX4 protein expression	[233]
SXBXW	Regulate the miR-144-3p/SLC7A11 signaling pathway	[238]
LHF	Upregulate of the SLC7A11/GPX4 pathway, activate the Keap-1/Nrf2/ARE antioxidant signaling pathway	[242, 243]
YXB	Activate the DJ-1/GPX4 signaling pathway	[245]

### Other products

Currently, research shows that iron metabolism in hemoglobin and its precursors or derivatives plays a crucial role in ferroptosis [246]. Heme, the precursor of hemoglobin, is a type of porphyrin compound that contains an iron ion. It plays a vital role in various biological processes, including oxygen transport, electron transfer, gene expression regulation, circadian rhythm, and signal transduction [42]. Heme is known to be generated in the mitochondrial matrix, and heme proteins and heme regulatory proteins are widely distributed throughout various subcellular compartments [44]. Given the hydrophobicity and oxidative properties of free heme, cells must possess specific pathways to safely transport heme from the mitochondria to the extramitochondrial environment. Recent studies have discovered that iron porphyrin serves as a biological target for hydrogen molecules, acting as a hydrogen molecular sensor and catalyst. Experimental results demonstrate that both the free and protein-bound forms of iron porphyrin selectively neutralize the highly toxic hydroxyl radical (·OH) through catalytic hydrogenation, thus mediating the antioxidant, anti-inflammatory, and anti-aging effects of hydrogen molecules. In oxygen-deficient microenvironments, such as hypoxic areas within tumors, iron porphyrin catalyzes the reduction of CO2 to CO, resulting in the in-situ mediation of CO signaling pathways and, ultimately, achieving anticancer and immune-regulatory therapeutic effects. Considering that iron porphyrin predominantly accumulates in mitochondria and red blood cells, this discovery effectively explains the mitochondrial regulatory effects and systemic inflammatory regulation effects of molecular hydrogen. These findings confirm CO as a downstream signaling molecule of hydrogen, providing a satisfactory explanation for the diverse biological effects of hydrogen that rely on features specific to the microenvironment of lesions [247]. Other research also reported that molecular hydrogen can activate the transcription factor Nrf2, alleviating oxidative stress through hydrogen-targeted porphyrins [248]. Early studies have discovered the selective clearance of highly oxidative and toxic hydroxyl radicals (·OH) within cells by hydrogen gas. In recent years, hydrogen molecules have been shown to possess significant biological effects in numerous oxidative stress and inflammation-related diseases, demonstrating broad-spectrum, safe, and effective anti-inflammatory/anticancer/anti-aging characteristics [249]. Moreover, hydrogen gas exhibits remarkably high tissue penetrability, surpassing physiological barriers that conventional drugs cannot overcome, such as the blood–brain barrier. This characteristic positions hydrogen gas as a promising therapeutic gas molecule with wide-ranging applications [250].

Hydrogen gas (H2) has demonstrated anti-inflammatory and antioxidant abilities in numerous clinical trials [251]. Current research findings suggest that H2 gas can protect the lungs and extrapulmonary organs from pathological oxidative stress stimuli [252]. Furthermore, H2 has been shown to regulate anti-inflammatory and antioxidant activities, mitochondrial energy metabolism, endoplasmic reticulum stress, immune system function, and cell death processes (including apoptosis, autophagy, pyroptosis, ferroptosis, and circadian rhythms), thereby exhibiting therapeutic potential for various systemic diseases [253–256]. Hydrogen sulfide (H2S), on the other hand, mitigates mitochondrial damage and ferroptosis by modulating the OPA3-NFS1 axis induced by doxorubicin-induced cardiotoxicity [257]. In the context of heart failure, hydrogen sulfide modulates iron metabolism, reducing oxidative stress levels in myocardial cells, inhibiting myocardial iron death, and protecting cardiac function in aging rats [258].

## Prospects

In recent years, with the rise of the concept of ferroptosis and the continuous deepening of research, it has been found that there are many mechanisms of ferroptosis, which have been greatly proven to be related to HF in aspects such as iron overload, lipid peroxidation, GSH–GPX4–ROS pathway, NADPH/FSP1/CoQ10 pathway, tumor suppressor gene P53, and oxidative stress. Moreover, single herbs and their extracts, traditional Chinese medicine (TCM) formulas, acupuncture and combined acupuncture-medicine have the characteristics of multiple pathways and targets for HF ferroptosis, providing convenience and reducing the burden for the majority of patients. The commonly used clinical treatments for HF, such as Pueraria, Ophiopogon, Astragalus, Chuanxiong, Salvia miltiorrhiza, Xinyang tablets, Yuxue Bi, Shenmai injection, Erchen decoction combined with Tao Hong Siwu decoction, Ditang decoction, and electroacupuncture, all exert their effects by inhibiting myocardial ferroptosis. However, since the research on ferroptosis in HF is still in its infancy, there are still many issues that need to be addressed in the future. Firstly, there are few and unclear studies on the mechanism and application of ferroptosis, and it is hoped that future research will strengthen the mechanism of ferroptosis in HF and further understand the pathological and physiological mechanisms of ferroptosis in HF. Secondly, there is little research on the use of TCM in the treatment of HF ferroptosis, and further clinical trials are needed to explore whether there are more TCMs that can exert myocardial protective effects by inhibiting ferroptosis, seeking more TCM treatments for HF. In addition, ferroptosis treatment of HF provides a theoretical basis for the formulation of prescriptions, new drug development, and academic hypotheses. Finally, it is hoped that ferroptosis can make new progress in the clinical treatment of other diseases.

## Data Availability

The data used to support the findings of this study are included within the article.
